# Developing a capacity building training model for public health managers of low and middle income countries

**DOI:** 10.1371/journal.pone.0272793

**Published:** 2023-04-21

**Authors:** Kritika Upadhyay, Sonu Goel, Preethi John

**Affiliations:** 1 Department of Community Medicine and School of Public Health, Post Graduate Institute of Medical Education and Research, Chandigarh, India; 2 Faculty of Education & Health Sciences, School of Medicine, University of Limerick, Limerick, Ireland; 3 Global Business School for Health, University College London, London, United Kingdom; Universitat Luzern, SWITZERLAND

## Abstract

**Background:**

The challenges faced by the low and middle-income countries (LMIC) in the field of public health management calls for the capacity building of qualified and trained public health managers in order to improve the effectiveness and efficiency of the health care delivery system. Most of the existing training programs for public health management are based in the settings of developed countries, which hinders their application in LMIC countries. The objective of this paper is to document the process of development and evaluation of a capacity building program for public health managers of various LMICs.

**Material and methods:**

A training program was developed using Kern’s six-step framework with several innovative learning and assessment methodologies and evaluation using Kirkpatrick training evaluation model. Delphi technique was used for program development.

**Results:**

This five to ten-day partly/fully funded six International Public Health Management Development Programs (IPHMDP) programs was conceptualized which enrolled 178 participants from 42 countries between years 2016 and 2019. Based upon the elaborative discussion in four rounds of Delphi technique, the problem and challenges faced by public health mangers and eight key competencies (viz. Leadership and governance, Project/ program planning, financial management, supply chain management, quality management, Human Resource management, monitoring and evaluation, and communication.) were identified. The group consensually agree upon a blended teaching methodology comprising of chalk and talk approach, inquiry based learning, participatory student based learning, small group instructions, gamification, project-based learning and field-based learning. There was a significant increase in participants’ knowledge score (P<0.0001) after all programs especially in the competencies of monitoring and evaluation, followed by project/ program planning, supply chain management and quality management. The majority (90%) submitted their action plan one week following the program, out of which 64% implemented their action plans within six months. A majority (54.7%) of participants were able to implement their learning once they went back by conducting similar training/ workshop/webinars in their settings.

**Conclusion:**

The comprehensive public health management program in LMIC settings strengthens the competencies of public health managers which can be replicated in similar settings across LMIC to mitigate diverse challenges in public health management.

## Introduction

Public health management workforce is defined as “the stock of all individuals engaged primarily in the improvement of the health of populations” [1]. These are a group of individuals who are engaged during a significant part of time in the improvement of the health system and create the condition within which people can be healthy [2]. With an increasing demand for quality healthcare in resource constraint settings, developing a skilled public health management workforce is the need of the hour to galvanize and optimize existing resources through their management competencies for achieving organizational goals [3]. The public health management workforce should have competencies including management, leadership, program planning and finance skills, system thinking, collaboration and partnership, communication and advocacy, governance, and organizational behavior [4,5]. These managerial competencies are essential to enhance the quality of health care services [6].

Capacity building in managing public health has been a mandate of various development organizations. For example, World Health Organization (WHO) has emphasized giving the right training to the right people to create an effective workforce for the delivery of healthcare [7]. Similarly, World Bank is helping countries build public health managers’ capacity through various complementary means, including training, technical assistance, studies, and equipment [8]. Besides, United States Agency for International Development (USAID) also mandates building the global health workforce’s capacity to strengthen the health system through various programs [9]. Few academic organizations and institutes are also involved in the capacity building of public health managers focusing on their country’s current health scenario. Joint Learning Initiative (JLI), a consortium of over 100 global public health leaders, has also emphasized strengthening the health workforce through training in public health management [10]. Goal17.9 of Sustainable Development Goals (SDG) also concentrated on capacity-building in developing countries, along with enhancing international support for effective implementation [11]. Many countries have focused on capacity-building of human resources in health to meet their primary care needs for the overall attainment of Universal Health Coverage [12–14].

Various developing economies have made significant progress in the last two decades in the realm of public health management. However, there has been the poor quality of service delivery, inefficient recruitment and retention system, lack of skill-based training of program managers leading to a critical shortage of trained human resources [15]. Further, an increasingly lesser focus has been on multiskilling of existing personnel, poor financial management, and poor governance [16]. The majority of these gaps are cited to be due to insufficient managerial competencies of public health managers at various levels of the health care system [17]. In developed countries, various short and long-term public health management programs are being conducted, such as Baylor University [18], George Washington University [19], and University of North Carolina [20]. The Robins Institute of Health Policy and Leadership program under Baylor University provides a masters in healthcare administration focusing upon installing knowledge specific to the healthcare industry. Similarly, the George Washington University Milken Institute School of Public Health (GWSPH) offers four certificate programs health administration, health policy, public health and, health corporate compliance. The UNC Gillings School of Global Public Health offers Masters in Public Health and health with leadership in practice concentration. Very few such programs are being offered in developing countries including India. The Institutes like the Indian Institute of Health Management Research (IIHMR) [21]; National Institute of Health and Family Welfare (NIHFW)–New Delhi [22], All India Institute of Medical Sciences (AIIMS)–Delhi [23], All India Institute of Hygiene and Public Health (AIIHPH)–Kolkata [24] offer short-term training programs for health personnel in key areas of health management. Additionally, multiple trainings for state governments in health management are conducted on special requests [25,26]. However, most of these programs are theoretical, extensively elaborative, and do not comprehensively cover various aspects in a single program [17]. Further, there are very few formal management training in the government sector before taking up any senior management positions [27]. Therefore, healthcare managers are designated to higher ranks based on their seniority without consideration of managerial and administrative capabilities.

In addition to this, most of the existing programs on public health management for Low and Middle income countries (LMIC), which have poorer health indicators as compared to high-income countries, are based in the settings of developed countries, which hinder their application in LMIC countries as they may theoretically train the participants in tools and techniques but fails to instill confidence in their application in respective countries. Svadzian A, et al. in their recent article has also advocated for having programs in LMIC settings and had put forth their perspective that “we should still ask why an African trainee must go to London or Boston to learn about control of sleeping sickness or malaria (and pay top dollars for such training)” [28]. The traditional colonial mindset in global health that expertise flows from North to South is being amply reflected in research, training, consultancy, and technical assistance, which has been cited as the “ripe for disruption” [28]. The LMIC can acquire better when exposed to similar settings through field visits and real-time case scenarios, replicating in their countries. Besides, they have more opportunities to be exposed to subject experts who have prior experience of working in LMIC settings. In addition, the participants also learn to manage their program within limited resources, which increases their knowledge about public health management and boosts their self-reliance to implement the learning in their country settings.

To address these gaps, there is a need to devise a comprehensive public health management program in LMIC settings that can build the competencies of public health managers of LMIC to manage the existing and emerging public health challenges effectively. After undergoing such a program, it is expected that the public health managers should be able to effectively contribute towards the overall improvement of the health care delivery system to achieve SDGs in limited-resource settings [29]. The objective of this paper is to document the process of development and evaluation of a capacity building program for public health managers for various LMICs.

## Material and methods

### Program settings

The Postgraduate Institute of Medical Education and Research (PGIMER) in Chandigarh, India, is an autonomous “Institute of National Importance” by an Act of Parliament of India functioning directly under the Ministry of Health and Family Welfare, Government of India. The institute is rated second best for medical education, training, and research in India. Department of Community Medicine, instituted in 1977, has been a pioneer in teaching, training and research activities. To address emerging challenges in public health training and education, the department was upgraded to School of Public Health (SPH) in the Tenth Five Year Plan of India (2002–06) [30]. The department aims to provide short-term training programs to national program managers and academicians for building their competencies in addressing public health challenges and strengthening efficiency within organizations and networks. Setting in the context of a Centre of Excellence in India with subject experts having rich experience and showcasing good and replicable practices from different regions is an ideal teaching ground for public health management for a global audience.

### Program goal and objectives

The five days (later became ten days after request from participants) biannual International Public Health Management Development Program (IPHMDP) was conceived in the year 2016 in technical collaboration with the developmental partners the International Against Tuberculosis and Lung Diseases (The Union) and Chitkara University, Punjab India, with an overall goal of building the capacity of public health managers working at the sub-national and national level of LMIC, who can improve management processes and practices within their organizations. The specific objectives were: change in the knowledge about various managerial competencies, satisfaction score of participants on program logistics and technical learning, and implementation of learning of program in their program setting.

### Program development

Before the actual conduct of the program, the program took three months for its development, including finalization of the course content, flow, methodology, course design, and faculty. Kern’s six steps framework guided the development process. The model was initially developed to provide a practical, theoretically sound approach to developing, implementing, evaluating and continually improving educational experiences in medical sciences; however, later it has been applied to several other health science curriculum [31–34]. The Kern framework focuses upon competency development based upon the need assessment of participants and has been amply demonstrated in many disciplines across different settings. The Six-Step approach for program development includes: 1) Problem identification, 2) Targeted needs assessment, 3) Goals and objectives, 4) Educational strategies, 5) Implementation, and 6) Evaluation and feedback.

The Delphi technique is a group consensus for developing new concepts [35]. Around 20 experts (5 senior academicians, 5 Public health manager/ program manager/ project manager, 5 clinicians, 5 Non-Governmental organization) were purposively selected based upon their knowledge and experience on the subject. The anonymity of experts during in the study were maintained. A total of 4 rounds were conducted. In order to identify the problem associated with competencies of public health manager and along with gaps in existing (PHM) courses on public health management which aims at inculcating key competencies, the experts reviewed existing programs on public health management across the globe in first round of Delphi technique. **“S1 Annexure”** Besides, the national health plan and SDG indicators of the participating countries were accessed and discussed. Thereafter list of competencies were presented to experts which was followed by extensive discussion on competencies required by a public health manager along with gaps in existing management and leadership programme across the globe. Then, the first author (SG), build consensus on the competencies to be included. In the second round the experts revisited the competencies. Based upon the competencies finalized by the experts, the tool (e.g.: knowledge questionnaire) to access them were developed. Further, the feedback questions about the program along with an action plan format (viz. a detailed plan outlining the action to be implemented at their workplace) were evolved. An agreement to list of questions in the various tools were reached after two rounds of Delphi technique.

In the third round, the goals and objectives of the program were developed. During this phase, the experts were instructed to compile prospective goal and objectives through the competencies finalized in earlier rounds, supplemented with literature search. The list of objectives were then presented in third round which was followed with discussions which led to their finalization. The discussion led to one goals and seven possible objectives. Thereafter these objectives were placed against the predefined competencies finalized during second round of Delphi technique. Two objectives were eliminated after detecting duplications whereas two were eliminated as they were quite narrow and just related to single competency desired for the program. During this round, the educational approach for conducting the program was also finalized wherein the expert shared their experiences with attending or conducting prior similar programs by them. Thereafter, during the fourth round, the program faculty along with dissemination policy was discussed and finalized.

### Program evaluation

The evaluation of the training program is done based upon Kirkpatrick training evaluation model. The 4 level model is described as ‘the worldwide standard for evaluation of training effectiveness’ and its use has been documented in several healthcare training programs [36–41]. Level 1 is Reaction, which analyzes the degree to which participants find the training favorable, engaging and relevant to their work, Level 2 is Learning, which estimates participants knowledge, confidence, and commitment based on their participation in the training, Level 3 is Behavior, from which participants told about application of program learning’s back in their job, and Level 4 is Results which analyses the participants satisfaction for various components of the program. All parameters were assessed through a 3 point Likert scale (1: poor, 2: average, 3: good). Further, qualitative feedback of all the programs was taken and presented as participants verbatim.

### Data management and analysis

Data on various tools of program (pre and post questionnaire for assessing knowledge on key competencies, feedback questionnaire, and action plan) was collected through Google Forms which was exported to MS Excel followed by. For each pre- and post-question, a correct response was scored “one” and an incorrect response was scored “zero.” The difference between pre and post mean competency score was calculated for all modules in the program. The feedback of program was assessed for parameters using the five-point Likert scale (1 = poor, 2 = average, 3 = good, 4 = very good, 5 = excellent). The analysis was done using the Statistical Package for Social Sciences (SPSS) version 20.

### Ethics statement

The institutional review board of Postgraduate Institute of Medical Education and Research exempted this study from ethical review (IEC-08/2020/ 1743).

## Results

A total of 6 IPHMDPs were conducted between the years 2016–2019, in which 178 participants from 42 countries attended the programs, 36 participants in first program, 27 in second, 35 in third, 24 in fourth, 24 in fifth and 32 in sixth. Out of total, there were 69 (38.7%) females and 109 (61.2%) males. Majority of participants were post graduates (n = 139,78%). Out of total, 87 were from India, and the remaining 91 participants were from Africa, South America, Asia, and Europe regions. Table 1 depicts the distribution of participants in various IPHMDPs.

**Table 1:** Distribution of participants of International Public Health Management Development Programs (2016–2019).

Demographic Characteristics		Program						
		1^st^ (2016)	2^nd^ (2016)	3^rd^ (2017)	4^th^ (2017)	5^th^ (2018)	6^th^ (2019)	Total
**Gender**	**Male**	27	20	19	14	14	15	109
	**Female**	9	7	16	10	10	17	69
**Qualification**	**Post Graduate**	30	26	21	24	9	25	139
	**Graduate**	6	1	14	0	15	3	39
**Profession**	**Academic**	16	8	4	8	2	4	42
	**Public health Managers**	14	18	14	11	14	18	89
	**Private/ NGO/medical officers/others**	6	1	17	5	8	10	47

**Fig 1:**
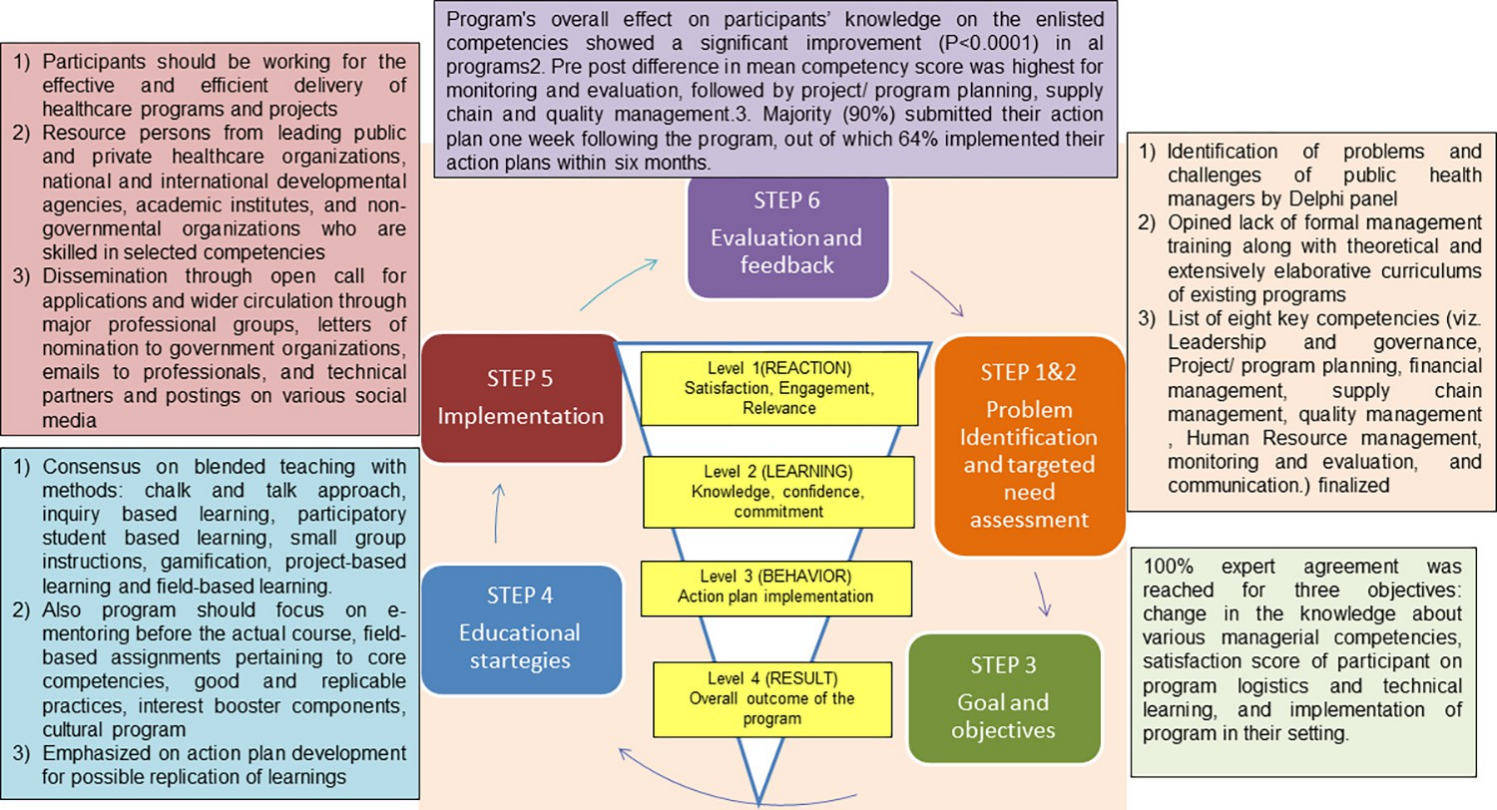
Conceptual framework for development and evaluation of International Public Health Management Development Program using Kern and Kirkpatrick framework.

The results of the study are presented through the Kern’s six steps and Kirkpatrick 4 step frameworks. Fig 1 represents the conceptual framework for development and evaluation of International Public Health Management Development Program (IPHMDP) using Kern and Kirkpatrick models.

### STEP 1 and 2: Problem identification and targeted needs assessment

The primary problems and challenges of public health managers identified by Delphi panel were the poor capacity to plan, impalement and evaluate programs or initiatives, inadequate supplies, and logistics, limited knowledge on financial management, poor health information management systems, lacking communication skills, lack of human resources along with limitations on effective leadership. Besides, the experts cited that lack of formal management training along with their theoretical and extensively elaborative curriculums are primary barriers to effective public health management. Based upon the consensus, it was felt that the creation of a well-designed public health management program is an opportunity to build management competencies of public health managers of LMIC for the overall improvement of health indicators of the country. A list of eight key competencies to address the felt need of participants were then finalized which were: leadership and governance, project/ program planning, financial management, supply chain management, quality management, human resource management, monitoring and evaluation, and communication. Based upon the competencies, a total of 8 modules were proposed for the entire program.

### STEP 3: Goals and objectives

Based upon the discussion during Delphi technique, 100% expert agreement was reached for three objectives which were: change in the knowledge about various managerial competencies, satisfaction score of participant on program logistics and technical learning, and implementation of program in their setting.

### STEP 4: Educational strategies

During third round of Delphi discussion, various teaching and learning techniques were discussed. The majority of Delphi members reached to the consensus on following methods: chalk and talk approach, inquiry based learning, participatory student based learning, small group instructions, gamification, project-based learning and field-based learning. The experts were in the agreement that selected participants should undergo e-mentoring before the actual course, where pre-reading material should be shared to familiarize them with the program’s content. During the course, the 2–3 day field-based assignments pertaining to core competencies of program were proposed in the nearby jurisdictions (of program settings) for demonstration of the good and replicable practices relevant and contextual for LMIC settings. For this, experts reviewed and finalized possible good and replicable practices based upon the listed competencies which can be demonstrated to the participants. (Table 2) The experts also opined that sharing of case stories and good practices by the participants developed in their country settings is of immense help to their peers in order to think of strategic solutions and help adapt to their context. The group also felt that interest booster components (management games, contests, quiz, etc.) should also be incorporated to refresh the participants during the program. Also, a cultural program should be arranged where the participants showcase the cultures of their country and meet informally for networking and collaborations. Each participant was also expected to develop an action plan after discussing it with the facilitators and implementing it within six months of the program. Finally the group consensually decided on a blended approach to learning which included a mix of all the selected approaches.

**Table 2:** List of competencies and good replicable practices.

Competencies	Good replicable practices
Leadership and governance	• Demonstration of an effective leadership model in making the state of Himachal Pradesh a tuberculosis free in the country. • Low cost anti rabies treatment: an innovation toward rabies free Himachal Pradesh
Project/ program planning	Demonstration of achievable indicator of any program implementation plan of a well performing district of Himachal Pradesh
Financial management	Illustration of financial management (annual budget plan and budget report) of a well performing district of Himachal Pradesh
Supply chain management	Demonstration of effective drug logistic system of a tertiary care hospital
Quality Management	• Demonstration of a skill laboratory for management of maternal and child health at Health and Family welfare training centre. • Demonstration of few PDSA cycles for quality improvement in a department of tertiary care hospital (e.g.: patient waiting time, breast feeding practices etc.)
Human resource management	• Participation in a training workshop and demonstration of effective training logistics, its delivery and evaluation mechanism • Demonstration of team at grassroots level for effective functioning of village health nutrition day
Monitoring and Evaluation	Demonstration of a digital program monitoring system using mobile application (e.g.: ANMOL application)
Communication	Demonstration of telemedicine facility at lowest health functionaries (health and wellness centre) for facilitating patient doctor communication

### STEP 5: Implementation

The expert’s selection dwelled upon the selection of participants and resource faculty along with dissemination strategies of the program. They consensually opined that the persons who have been working for the effective and efficient delivery of healthcare programs and projects should be the participants. A written commitment about replication of the program’s learning was sought to be taken prior to the selection of participants. The resource persons were selected from leading public and private healthcare organizations, national and international developmental agencies, academic institutes, and non-governmental organizations working at the grass-root level, who have prior experience and expertise in delivering the competencies of the program.

For the national courses (first, second and fourth), the selection of participants was sought to be made one-two month prior to the program through an open call for applications and wider circulation through major professional groups, letters of nomination to government organizations, emails to professionals, and technical partners and postings on various social media handles including the institute websites. The international participants from LMIC were nominated by their respective governments through the Indian Technical and Economic Cooperation (ITEC) programme under the Ministry of External Affairs, Government of India. These programs are designed to build the capacity of professionals from 161 countries in Asia, Africa, East Europe, Latin America, the Caribbean, and Pacific and Small Island countries in demand-driven emerging areas of shared concern along with the development of mutual cooperation and partnerships. A group of 20–40 participants were pronounced to be selected by taking representations from different states of country/other countries, gender, academic profile, organization, and experience.

The evaluation of the training program was also done in four stages of Kirkpatrick’s model viz. reaction, learning, behavior and results (Table 3).

**Table 3:** Evaluation of various items of IPHMDP based of Kirkpatrick evaluation model.

Kirkpatrick Domain^a^	Items of IPHMDP (n = respondents)	Rating		
		Poor n (%)	Average n (%)	Good n (%)
Reaction	Prior information about the program logistics (178)	4(2.5)	14(7.7)	160(89.8)
	Availability of venue and necessary facilities (177)	7(2.5)	12(7.3)	158(90.1)
	Flow and content of the presentations (178)	3(4.2)	10(7.0)	165(88.8)
	Pace and the sequencing of the sessions (178)	3(1.7)	8(5.4)	167(92.9)
	Mix of teaching methodologies (178)	3(1.7)	14(7.8)	161(90.6)
	Active learning (178)	1(1.7)	7(8.4)	170(89.9)
	Appropriateness of participants (178)	3(1.7)	4(4.7)	171(93.6)
	Experienced faculty (178)	4(0.8)	5(3.9)	169(95.3)
	Effective support team (178)	6(2.5)	2(3.2)	170(94.2)
	Prior knowledge about "takeaway" of the program (177)	4(2.5)	13(14.6)	160(82.9)
	Stated objectives met during program(178)	4(1.7)	6(4.7)	168(93.6)
	Relevant to job responsibilities (178)	4(2.5)	26(5.0)	148(92.5)
	Adequate resource material provided (178)	4(2.5)	9(0.8)	165(96.6)
Learning	Increased familiarization with state of the art/ good practices (178)	3(1.7)	13(4.2)	162(94.1)
	Strengthened knowledge and skills (178)	3(1.7)	8(2.3)	167(96.1)
	Overcome language & other barriers (178)	3(1.7)	15(7.4)	160(90.9)
	Developed networks & relationships (178)	3(1.7)	8(4.3)	167(94.0)
	Increased and skills for countering public health problems (178)	4(2.5)	9(4.8)	165(92.7)
	Intend to use learning from the program in settings work (178)	3(2.5)	8(2.6)	167(94.9)
	Recommend program to colleagues (177)	4(3.3)	1(0.9)	172(95.8)
Result	Overall rating of the program (177)	4(2.5)	8(4.6)	165(92.9)

^a^ The behavior component of Kirkpatrick domain was evaluated based upon the action plan implementation report submitted by participants.

### Level 1: Reaction

The satisfaction regarding the logistics of the program and information sharing with them prior to the program was reported as “good” by 160(89.8%) participants, whereas 14(7.7%) and 4(2.5%) rated it as ‘average’ and ‘poor’ respectively. 158 (90.1%) responded that the venue had all requisite facilities and necessary comforts. The presentations prepared and the pace and the sequencing of the sessions was reported as “good” by majority of the participants (n = 165, 88.8%) and (n = 167, 92.9%) respectively.

The following quotes express the participants’ opinions regarding satisfaction or favorable component of reaction.

- *The planning and designing of the program as wonderful*. *It was time bound and systematically managed but can be extended to 2 weeks for better learning*
- *Learning environment through management games and videos during the break was very innovative*. *They helped us to practically apply the learnings at our workplace*.
- *Accommodation*, *transport*, *training hall were very well organized*. *The stay was very pleasant & comfortable*

In engagement, 161(90.6%) of the participants responded ‘good’ on the effectiveness of the mix of methodologies and 170(89.9%) participants said they were engaged in active learning. 171 (93.6%) agreed to the appropriate number of participants in the program, whereas 169(95.3%) and 170(94.2%) responded faculty and support team of the program were effective.

Regarding the program’s ‘relevance’, most participants opined that they had prior knowledge regarding the takeaway" from the program. 173 (97.3%) respondents agreed to the relevance of program to their work setting while (n = 178, 100%) agreed to the fact that such training programs should be incorporated into their routine practice for professional development. 168(93.6%) participants said the program met its stated objectives, and 148(92.5%) believed the program was relevant to their job responsibilities. The majority, 165 (96.6%), agreed that the resources/material provided would be helpful in their program settings.

The following quotes express the participants’ opinions regarding ‘engagement’ component of reaction

- *The speakers from various backgrounds were very good*, *highly experienced and motivating*. *They have well-prepared presentations*, *case scenarios*, *and group activities*.
- *A systematic approach of organizers*, *blended with personal attention was outstanding*.
- *Perfect blend of innovative teaching methodologies was the USP of the program*. *Action plan and Field tours are real learning sources in the program*
- *The program helped in my personal development & my career and will be role model to other programs in India and Globally*.

### Level 2: Learning

167(96.1%) participants opined that they strengthened the knowledge and competencies in the selected area of public health management. 160(82.9%) participants felt that the program increased their familiarity with ‘state of the art and good practices’ in public health management. The confidence was reported by 160 (90.9%) participants who overcame language & other barriers for better understanding during the program. The majority (n = 167, 94%) agreed to the fact that the programs helped them in developing networks & relationships with other participants. Also, there was a significant increase (n = 165, 92.7%) in their knowledge and skills for countering the public health problems in the country after attending the course. The commitment to apply the learning’s at work was quoted by 167(94.9%) participants, whereas 172(95.8%) assured to recommend the program to their colleagues on going back to their respective countries.

**Fig 2:**
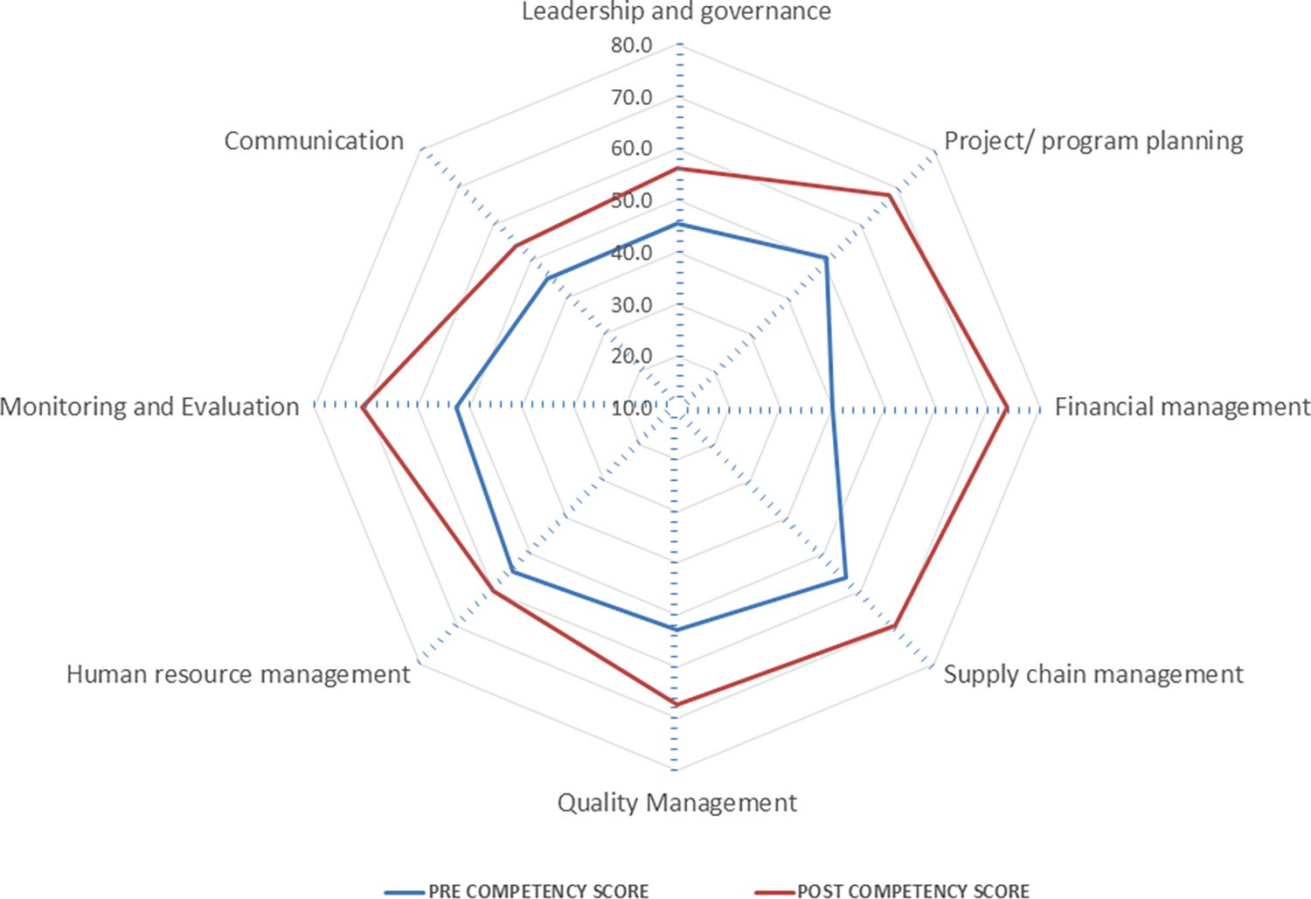
Pre post mean competency score of modules in International Public Health Management Development Program.

The learning was also evaluated through pre post-examination conducted at the beginning and end of the program. The figure shows the difference of mean competency score of the participants attended 6 IPHMDPs. (Fig 2) There was a statistically significant difference in the knowledge regarding all the key competencies after attending the programs. (Table 4). The pre post difference in mean competency score was highest for financial management, followed by monitoring and evaluation, supply chain management, project/ program planning and quality management. The program’s overall effect on participants’ knowledge on the enlisted competencies showed a significant improvement (P<0.0001) in all the programs (Fig 2 and Table 5).

**Table 4:** Change in level of competency of participants of International Public Health Management Development Program.

Competency	Pre (Mean ±SD)	Post (Mean ±SD)	95% Confidence Interval of the Difference		P Value
			Lower	Upper	
Leadership and governance	1.78±1.6	2.32±2.0	-1.05221	-0.01445	0.046
Project/ program planning	1.50±0.7	1.91±0.4	-0.77880	-0.03787	0.037
Financial management	1.20±0.3	1.85±0.6	-1.25912	0.08912	0.076
Supply chain management	1.99±0.9	2.45±1.1	-0.88792	-0.02542	0.042
Quality Management	1.57±0.4	2.12±0.3	-0.91250	-0.18750	0.011
Human resource management	1.61±0.5	2.01±0.7	-0.75296	-0.04704	0.033
Monitoring and Evaluation	1.82±0.5	2.57±0.5	-1.25825	-0.24509	0.012
Communication	1.26±0.5	1.56±0.6	-0.50483	-0.10183	0.012

**Table 5:** Impact of public health management program on participant’s knowledge score.

Program	Pre Test (Mean ± SD)	Post Test (mean ± SD)	T-statistic	DF	p value
**Program 1 (n = 25)** ^a^	11.35 ± 0.8	12.6 ± 0.7	5.174	48	<0.0001
**Program 2 (n = 20)** ^a^	16.19 ± 0.6	20.82 ± 0.5	26.511	38	<0.0001
**Program 3 (n = 30)** ^a^	18.57 ± 0.6	21.21 ± 0.7	15.684	58	<0.0001
**Program 4 (n = 40)** ^a^	16.71 ± 1.4	22.43 ± 1.7	16.427	78	<0.0001
**Program 5 (n = 30)** ^a^	10.98 ± 0.5	15.27 ± 0.5	33.23	58	<0.0001
**Program 6 (n = 30)** ^a^	13.07± 0.7	14.53 ± 0.7	8.078	58	<0.0001

^a^ n denotes the number of questions asked during pre-post test.

The following quotes express the participants’ opinions: regarding the ‘learning’ level of Kirkpatrick model:

- *The content and flow of presentations were simple and understandable*. *Pre-read material and reading materials were very informative and interesting*
- *Interactive sessions*, *including role-plays and games were very productive*. *They added flavours to teaching*
- *Practical information imparted was of utmost relevant to our work areas*.

### Level 3: Behaviour

160(90%) of the participants submitted their action plan about one topic following one week of the program in the behavior level. Of these, 115(64.6%) reported the implementation of their action plans within six months. The majority 63(54.7%) participants conducted similar training/ workshop/webinars in their settings, while rest were engaged in health promotional activities 4(3.47%), collaboration 7(6.08%), and implementing knowledge learnt in academic/program settings 24(20.8%) (Table 6).

- *We learned the theoretical concepts of public health management and now we can implement them with determination in our organization*.
- *The program helped me in designing and implementation of health activities in our day to day settings*

**Table 6:** Output of IPHMDP received through Action plan submitted by participants.

Output	Number (%) (n = 115)
Teaching and training/ workshop/conference /webinar	63 (54.75)
Implementing knowledge and ideas in academic settings	24 (20.8)
Implementing knowledge and ideas in program settings	17 (14.7)
Other academic collaboration / new setups, planning of camps etc.	7 (6.08)
Health promotional activities	4 (3.47)

### Level 4: Result

A total of 165(92.9%) rated the program as “good”, 8(4.6%) rated it as ‘average’ and 4(2.5%) reported it as ‘poor’.

## Discussion

Public health management training is often being overlooked by organisation of LMIC but has shown a significant difference in operational efficiency, staff satisfaction and effectiveness, and quality improvement [42,43]. The present study provides an overview of public health management development programs conducted in an LMIC setting with an effort to build the capacity of public health managers. The current public health management program in LMIC settings successfully built the capacity of the public health managers by carefully selecting diverse participants from various fields, which were similar to another study where varied category of health workforce were trained through an almost similar modality [44]. We found that such a comprehensive public health management program implemented in LMIC settings strengthens the competencies of public health managers, which can further be replicated in similar settings to mitigate diverse challenges in public health management.

The study has several strengths. Firstly, a well-designed comprehensive curriculum enabled participants to develop competencies for designing, implementing, monitoring, and evaluating program/ project operations in their respective countries. It equipped participants in leadership competencies and on appreciating gaps in current health scenarios in their countries and envision future trends in health care management for effective decision making. Second, the two validated frameworks (Kerns six-step framework and Kirkpatrick training evaluation) were used to diagnose the existing problem and need of LMIC and further evaluate the implemented program. Third, the continuous quality improvement of the subsequent training programs was done by incorporating feedback of stakeholders. For each programme appropriate changes were incorporated. For example, the feedback from participants and alumni lead to increased number of training days from 5 to 10 days. Also, e-mentoring was proposed in later programs to the participants. Further, the change based on feedback from resource persons leads to the development of country-specific case studies as a resource material; which lead to cross culture learning and the sharing of ideas and strategies. This continuous improvement through the course amplified the relevance of the program and the sustainability of the program. Fourth, the output-oriented curriculum lead to application of learning’s in their respective settings which boosted the confidence of the participant, eventually leading to increased performance and productivity of the organization.

The study’s key findings were its uniqueness in production and implementation of a need-based curriculum for public health mangers demonstrating enhanced satisfaction of training, relevance, and adequacy at LMIC settings. This curriculum was purposively developed to let the participant have an exposure of adult learning methodology along with application-based learning. The schedule not only demonstrated the development process of the curriculum which could be implemented by other LMIC, but additionally present findings regarding participant engagement, satisfaction, and relevance. The highlight of current capacity building program in terms of information dissemination, selection of participants, selection criteria was found similar to the study by Ramsay et al. [45]. The training format used in the study was similar to another capacity-building program by Kumar AMV et al, as both included lectures, break-out mentor groups, and plenary sessions [46]. The program was assessed through pre-post evaluation and its implementation post-program similar to another study by K. MacVarish et al. [44]. Session and the overall feedback was collected using questionnaires covering quantitative and qualitative aspects about the background information of the participants, importance of program in their job profile; the significance of different methods used for teaching overall perception of the course its usefulness and application of knowledge from the courses to their current job, similar to the study by Zackoff [47]. Similar to other programs, the current program was evaluated on the parameters reflecting adequacy of the content, relevancy, achievement of aim and objectives, flow of module, appropriateness of teaching methods, quality of mix of teaching methods, participation of participants, module expectations [44,46]. The distinguishing feature of the study amplifying the voice of public health management training for LMIC managers has been a comprehensive evaluation and adult learning pedagogy developed and implemented in LMIC setting. This was also cited by Svadzian A, et al in their recent article, they put forth their perspective that “we should still ask why an African trainee must go to London or Boston to learn about control of sleeping sickness or malaria (and pay top dollars for such training)? [28]. A robust conceptual framework of using the Kerns Framework of curriculum development and Kirkpatrick framework for training evaluation has been used in the current study as mentioned by Zackoff et al, Haller et al. [48,49]. Overall, the participants responded positively to the program and agreed the program fully met the aim and objectives.

The model used in the study has a few limitations. The model measures what participants have learned, but does not measure the involved interest and motivation factors behind the learning. During subsequent programs the perceived motivation of participants could be sought. The model does not talk about the impact of skills demonstrated during the program or the program’s long-term effects on change in moral behavior, or the program’s financial impact. We are working upon to access the long term changes on their moral behavior and program outcomes. Besides the model, the study also has few limitations. The current study has a relatively small number of participants in a particular geographical region and health system context. This can be eliminated by enrolling an almost similar number of participant’s different WHO regions. Further the participants demanded translating lectures in their respective languages, which was not feasible due to the increased cost. In future, recorded lectures can be uploaded on some modern IT software where different languages are available The other challenge was the dropout rate from a long-term follow-up on the action plan implementation. Without a separate financial support in place, it becomes difficult to continuously follow up with the participants in terms of long-term outcomes, post-program implementation of the learning’s from the program. The effort can be put into developing the curriculum having an online version of the course would help to reach more people across boundaries.

## Conclusions

The current public health management program was effectively developed and implemented in LMIC settings to enhance the knowledge, competencies, and self-sufficiency of global participants of LMIC settings for strengthening their country’s health systems. The organizations, government, and non-government should carefully adopt the interventions in the program for individual and organizational growth based upon their contextual settings. The study also suggests a few recommendations and way forward for conducing similar programs. First, the LMIC based public health management programs will have a more significant impact from an implementation point of view due to the application of contextual learning back at the workplace. Second, the curriculum of the public health management program should be integrated with graduate and postgraduate education systems in strengthening public health management. Third, the integration of PHM alumni for training the upcoming programs will enhance the competencies and confidence of the participants. Fourth, the program’s reach can be increased by providing an online platform to facilitate the participation of more countries who cannot attend because of time and resource constraints. Fifth, collaboration with various stakeholders-nationally and internationally is needed for increasing visibility and robustness to the program. The financial or/and technical support for its sustenance is required to strengthen global health systems.

## Supporting information

S1 Annexure Top 20 global public health management courses. (PDF)
