# Supplementary material for: Developing a capacity building training model for public health managers of low and middle income countries
Source: PLoS One. 2023 Apr 21;18(4):e0272793. doi: 10.1371/journal.pone.0272793 (PMC10121058; doi:10.1371/journal.pone.0272793)
Supplement: S1 Annexure — (PDF) [file pone.0272793.s001.pdf]

Annexure 1: Top 20 global Public health management courses										
**S. No.**	**Institute**	**Ownership (Public/ Private)**	**Course Name**	**Duration (in Years)**	**Type of course (Degree/ Diploma)**	**Teaching approach/ Teaching methodology**	**Teaching content**	**Key skills/Competencies**	**Evaluation Criteria (Credit Hours)**	**Link**
01.	Harvard University, Cambridge, United States	Private	Master in Health Care Management (MHCM)	02 year	Degree	Interactive, Case-Based Learning, Peer Group Collaboration	https://www.hsph.harvard.edu/mhcm/schedule-curriculum/	Managerial skills, Leadership skills	42.5 Credits	https://www.hsph.harvard.edu/admissions/degree-programs/#MHCM
02.	Johns Hopkins University, Baltimore, United States	Private	Master of Science in Health Care Management	01 year	Degree	Class room teaching and Project-based	https://carey.jhu.edu/programs/master-science-programs/health-care-management	Business skills, Experiential learning, Leadership skills	36 credits	https://carey.jhu.edu/programs/master-science-programs/health-care-management

0 3.	London School of Hygiene and Tropical Medicine, London, United Kingdom	Public	MSc Health Policy Planning & Financing	01 year	Degree	Lectures, small group seminars, practical, and group work with peers.	https://www.lshtm.ac.uk/study/courses/masters-degrees/health-policy-planning-financing#structure	National health policies planning and financing	185 Credits	https://www.lshtm.ac.uk/study/courses/masters-degrees/health-policy-planning-financing
0 4	Erasmus University Rotterdam, Rotterdam, Netherlands	Public	European Master in Health Economics and Management	02 years	Degree	Lectures, small group seminars, practical, and research	https://www.eur.nl/en/eshpm/master/european-master-health-economics-and-management/during-your-studies	Health economics and management in health policy, businesses and healthcare organisations.	120 Credits	https://www.eur.nl/en/eshpm/master/european-master-health-economics-and-management
		-Do-	Health Care Management	01 years	Degree	Lectures, small group seminars, practical, and exchange program	https://www.eur.nl/en/eshpm/master/health-care-management/curriculum	Innovation, Strategic problem-solving, analytical, Technical, management skills	60 Credits	https://www.eur.nl/en/eshpm/master/health-care-management
0 5	University of Minnesota Twin Cities, Minneapolis, United States	Public	Healthcare Management Certificate	8-10 months	Certificate course	Classroom teaching	https://www.sph.umn.edu/academics/degrees-programs/certificate/healthcare-management/curriculum/	Leadership, decision making, resource management , financial management, management in healthcare organizations	12 credits	https://www.sph.umn.edu/academics/degrees-programs/certificate/healthcare-management/

06	Monash University, Melbourne, Australia	Public	Health Management	1.5 years	Degree	Classroom and field based teaching	Eight core and three elective units https://www.monash.edu/study/courses/find-a-course/2022/health-management-m6008?international=true#course-structure-3	Leadership and management, financial management, quality improvement, health policy, law for health systems, principles of health systems	48 Credits	https://www.monash.edu/study/courses/find-a-course/2022/health-management-m6008?international=true#overview-1
07	University of New South Wales, Sydney, Australia	Public	Master of Health Leadership and Management	1+(1) Year (s)	Degree	Lectures, small group seminars, practical, and field based learning.	https://www.unsw.edu.au/study/postgraduate/master-of-health-leadership-and-management?studentType=Domestic	Strategic planning, policy development, change management, financial management, health economics, evidence-informed decision-making, clinical governance, risk management, quality improvement, integrated care, patient safety, value based healthcare.	48 Credits	https://www.unsw.edu.au/study/postgraduate/master-of-health-leadership-and-management?studentType=Domestic

08	Brandeis University, Waltham, United States	Private	Master of Science in Global Health Policy and Management	09 months	Degree	Classroom teaching and assignment	https://heller.brandeis.edu/courses/classes/2019/Fall/2900/all	Financial management, health systems strengthening, health system and policy	36 credits	https://heller.brandeis.edu/global-health-masters-program/curriculum/index.html
09	University of Essex Online Colchester United Kingdom	Public	MSc International Healthcare Management	02 years	Degree	Multimedia lecture casts, short videos, animation, audio recordings, infographics, short tests and mini-assignments, discussion forums, eLibrary of textbooks and academic journals	https://online.essex.ac.uk/courses/msc-international-healthcare-management/#coursestructure	Leadership skills<br>Public policies<br>Planning for effective and efficient delivery of healthcare services<br>Concept of evidence-based management practice	180 credits	https://online.essex.ac.uk/courses/msc-international-healthcare-management/#overview

10	Ohio State University Columbus, Ohio, USA	Public	PhD Health Services Management and Policy -	3-4 years	Degree	Research learning programme	https://cph.osu.edu/prospective-students/phd/health-services-management-and-policy	Leadership skills, health care management, health policy analysis, health services research, quality-of-care measurement and improvement, health finance and health economics	80 credits	https://cph.osu.edu/prospective-students/phd/health-services-management-and-policy
11	University of Iowa, Iowa City. USA	Public	Ph.D. in Health Services Research and Policy.	3-4 years	Degree	Research learning programme	https://catalog.registrar.uiowa.edu/public-health/health-management-policy/health-services-policy-phd/	Management of health care organizations, formulation and implementation of health care policy, improving access to health care services, financing health care services, and evaluating the quality and outcomes of health care services.	72 Credits	https://www.public-health.uiowa.edu/hmp-overview/

12	St. Louis University St. Louis, United States	Private	Master of Health Care Management (M.H.C.M.) degree	16 months	degree	Multimedia lecture casts, short videos, audio recordings, short tests and mini-assignments, discussion forums, and field visit.	https://www.slu.edu/public-health-social-justice/education/graduate/health-care-management-master-of.php	Leadership, management, critical thinking and analysis, communication	41 credit	https://www.slu.edu/public-health-social-justice/education/graduate/health-care-management-master-of.php
13	University of Pittsburgh Pittsburgh, United States	Public	Healthcare Management Bachelor of Science	02 years	degree	Multimedia lecture assignments, discussion forums, and field visit.	https://www.greensburg.pitt.edu/academics/majors-minors/healthcare-management#coursedesc	Healthcare delivery, financial management and managed care, healthcare policy, law and ethics	120 credits	https://www.greensburg.pitt.edu/academics/majors-minors/healthcare-management#coursedesc
14	Virginia Commonwealth University, Richmond, United States	Public	Health Care Management Concentration Summer - Executive Format Weekend Delivery	03 Months	Certificate	Comprehensive tuition, project work, assignments	https://business.vcu.edu/media/business/docs/2022%20Health%20Care%20Management%20Concentration%20Overview.pdf	Policy issues and information technology support, business and financing of health care, and insurance	9 credit	https://business.vcu.edu/academics/mba-options/health-care-management-concentration/

15	University of Florida, Gainesville, FL, United States	Public	Population Health Management	02 years	Degree	Lectures, practical, group work and project work.	https://mph.ufl.edu/wordpress/files/2021/08/Population-Health-Management-Curriculum-F21.pdf	Leaders, healthcare manager and policy developer in public health	48 credits	https://mph.ufl.edu/about/concentration-options/population-health-management/
16	University of Toronto, Toronto, Canada	Public	Master of Science (MSc) in Health Policy, Management and Evaluation	03 years	Degree	Lecture, research practicum, and assignments	https://www.sgs.utoronto.ca/programs/health-policy-management-and-evaluation/	Clinical Epidemiology and Health Care Research, Quality Improvement and Patient Safety, Leadership	3.0 full-course equivalents (FCEs) Credit/No Credit basis	https://www.sgs.utoronto.ca/programs/health-policy-management-and-evaluation/
17	University of Washington, Campus Parkway, Seattle, WA, USA	Public	Leadership and Management in Health	03 Months	certificate	Video lectures, readings, discussion forums, quizzes, and assignments	https://edgh.washington.edu/courses/leadership-management-health	Organizations and manage people, practical leadership and management skills	8-12 weeks Continuing Education Units (CEUs)	https://edgh.washington.edu/courses/leadership-management-health

18	University College London, London United Kingdom	Public	Global Healthcare Management (Leadership) MSc	01 year	degree	Written exams, Coursework essays, Presentations, Business project,Research project	https://www.ucl.ac.uk/prospective-students/graduate/taught-degrees/global-healthcare-management-leadership-msc	Learning about global differences and interconnections, quality improvement, patient safety and workforce management	180 Credits	https://www.ucl.ac.uk/prospective-students/graduate/taught-degrees/global-healthcare-management-leadership-msc
19	Imperial College London, London, United Kingdom	Public	MSc International Health Management	01 year	degree	Video lectures, readings, discussion forum, guest lectures or alumni and careers events	https://www.imperial.ac.uk/business-school/programmes/msc-international-health-management/programme/#summer-projects	Entrepreneurship, Health Management and Innovation Studies.	90 credits	https://www.imperial.ac.uk/business-school/programmes/msc-international-health-management
20	University of Cambridge, Trinity, Cambridge, United Kingdom	Public	Health Care Management	18 months	degree	Recorded presentation, live lectures, practical exercises, project work	https://boston.cambridgecollege.edu/degree/health-care-management-1	leadership roles practical and operational needs	120 Credits	https://boston.cambridgecollege.edu/degree/health-care-management-1
